# Emerging Transcatheter Therapies for Valvular Heart Disease: Focus on Mitral and Tricuspid Valve Procedures

**DOI:** 10.3390/life14070842

**Published:** 2024-07-02

**Authors:** Nikolaos Ktenopoulos, Odysseas Katsaros, Anastasios Apostolos, Maria Drakopoulou, Grigorios Tsigkas, Constantinos Tsioufis, Periklis Davlouros, Konstantinos Toutouzas, Antonios Karanasos

**Affiliations:** 1 First Department of Cardiology, National and Kapodistrian University of Athens, Hippokration General Hospital of Athens, 11527 Athens, Greece; nikosktenop@gmail.com (N.K.); odykatsaros@gmail.com (O.K.); anastasisapostolos@gmail.com (A.A.); mdrakopoulou@hotmail.com (M.D.); ktsioufis@gmail.com (C.T.); ktoutouz@gmail.com (K.T.); 2 Department of Cardiology, Patras University Hospital, 26504 Patras, Greece; gregtsig@upatras.gr (G.T.); pdav@upatras.gr (P.D.)

**Keywords:** transcatheter therapies, valvular heart disease, mitral regurgitation, tricuspid regurgitation, transcatheter mitral valve replacement, transcatheter tricuspid valve replacement, transcatheter edge-to-edge repair

## Abstract

The emergence of percutaneous treatment options provides novel therapeutic alternatives for older and feeble patients who are at high risk for any surgical procedure. The purpose of our review was to offer an up-to-date analysis of the rapidly expanding field of percutaneous technologies for mitral, tricuspid, and pulmonary procedures. Edge-to-edge repair is an established treatment for secondary mitral regurgitation (MR), while transcatheter mitral valve replacement is a potential and expanding option for managing both secondary and primary MR. However, additional advancements are necessary to enhance the safety and feasibility of this procedure. Transcatheter tricuspid intervention is an emerging option that was conceived after the success of transcatheter procedures in aortic and mitral valves, and it is currently still in the early stages of advancement. This can be attributed, at least in part, to the previously overlooked effect of tricuspid regurgitation on patient outcomes. The development of edge-to-edge repair represents the forefront of innovations in transcatheter procedures. There is a scarcity of data about tricuspid annuloplasty and replacement, and further study is necessary. Transcatheter mitral, tricuspid, and pulmonary procedures show prospects for the future, while their role in clinical practice has not been definitively established.

## 1. Introduction

The first transcatheter heart valve (THV) implantation in 2002 [1] paved the way for the percutaneous valve therapies to witness rapid technological progress. This has generated a growing interest in valvular heart disease (VHD), which is an important group of diseases with increasing clinical reverberations and substantial financial distress [2]. The occurrence and frequency of VHD has expanded, mostly as a result of the rising life expectancy of the population [2]. Expansion in the surgical field of VHD, and currently in the transcatheter VHD procedures, are changing the way we treat patients at a gallop by providing less invasive treatment choices. Furthermore, advancements in imaging technology have yielded more accurate information on the structure and function of heart valves [3]. This has facilitated superior unification of images for scheming and managing procedures [3]. The acknowledgment of the relevance and efficacy of catheter-based treatments for VHD has also heightened enthusiasm for them and their implications [4].

VHD refers to a condition that affects any of the four valves of the heart. Since its inception in 2002 [1], transcatheter aortic valve implantation (TAVI) has significantly transformed the medical treatment of aortic stenosis (AS). Due to advancements in relevant equipment and procedural methods, TAVI has emerged as the primary treatment alternative for individuals with severe symptomatic AS [5], routinely performed as an elective procedure in stable patients and in some cases as an urgent one [6]. The implementation of various advancements, including the transition from conscious sedation to a single arterial access perspective [7], the adoption of novel implantation methods, and the utilization of innovative technologies like intravascular lithotripsy [8,9], as well as the improvement of THV appliances, has led to a significant decrease in complications and an enhancement of related results [9,10,11]. Evidently, recent data from important trials suggest TAVI to be on par with, or even better than surgery, paving the way for its use in younger and lower-risk patients with different AS categories [12,13,14]. In fact, for older patients with higher surgical risks, TAVI has become the preferred choice, while for younger patients, there are still open issues, with a notable drawback being the limited lifespan of the biological material utilized for the valve leaflets, which is the same for both transcatheter and surgical valves. This raises questions regarding the long-term outcomes of these valves and a potential need for re-intervention later in a patient’s lifetime, with this being the main reason for the use of mechanical valves in younger patients undergoing aortic valve replacement (AVR). Therefore, TAVI implantation in younger patients should account for the potential need for repeat operations, which are difficult due to the presence of adhesions between the stent and tissue and entail a mortality risk of 15–20% [4,15], or the need for a transcatheter valve-in-valve (ViV) implantation. This is now a component of the discourse on the patient’s age at the first implantation of the biological valve and the subsequent long-term treatment plan derived from it.

As a result, the latest guidelines from the European Society of Cardiology (ESC) recommend TAVI as the primary treatment option for patients aged 75 years or older with severe AS [15]. Although currently surgical aortic valve replacement (SAVR) is the preferred treatment for patients with native aortic regurgitation (AR) without AS [15], ongoing studies are exploring the potential use of TAVI with innovative THV systems to address the unsettled challenges, such as the JenaValve Trilogy. In the ALIGN AR trial the researchers demonstrated the safety and efficacy of this THV in high surgical risk patients with symptomatic, severe AR [16].

Moreover, it has been observed that mitral regurgitation (MR) is seen in more than 1% of the populace in Western countries who are over 70 years old and is linked to elevated mortality rates [2]. Although the surgical treatment of the mitral valve (MV) accounts for 10% of all surgeries [15], it is still inadequate in managing the problem of transcatheter treatment of MV conditions [17]. In addition, the prevalence of substantial tricuspid regurgitation (TR) in individuals over the age of 70 exceeds the 5% of the populace, with an accountable link between moderate or severe TR and worse long-term survival rates [2,18,19]. However, surgical or percutaneous procedure is rarely used to treat it [4]. It is the most frequent abnormality of the tricuspid valve (TV), and in the majority of the occasions (90–95%), TR is functional, caused by factors such as enlargement of the right ventricle or atrium, rather than by a primary cause, such as trauma, radiation, or endocarditis [20]. The distinguished invasiveness of surgical treatment and the inadequate contemplation of MR and TR in terms of their actual effects on patient survival and symptoms might be contributing factors to the lack of effective interventional treatment. Therefore, these components are potential areas of focus for further study.

Furthermore, in view of the pulmonary valve (PV), tetralogy of Fallot (TOF) is the predominant cyanotic congenital heart disease (CHD) and it is commonly addressed in a similar manner to other CHDs [21]. In the past, surgery was commonly used for PV replacement (PVR) and to alleviate right ventricular (RV) outflow tract (RVOT) deterioration [22]. In the year 2000, Bonhoeffer et al. performed the inaugural implantation of a bovine THV in an ovine model and subsequently in a 12-year-old boy [23]. In 2010, the Melody transcatheter PV, developed by Medtronic Inc. in Minneapolis, received Food and Drug Administration (FDA) acquiescence for mercantile use in malfunctioning PV conduits in the United States. Further research led to the compliance of the Sapien XT valve (Edwards Lifesciences LLC, Irvine, CA, USA) as a THV for cases with dysfunctional PVs [24].

Since the role of TAVI in the management of severe AS is established and very well studied, this review aims to provide a comprehensive summary of all currently present and developing transcatheter treatments for VHD in the MV, TV, and PV.

## 2. Transcatheter Interventions for Mitral Valve Disease

### 2.1. The Evolving Burden of Mitral Valve Disease

The predominant factor leading to mitral stenosis (MS) is mostly associated with rheumatic disease (RHD), which may develop in individuals who have had rheumatic fever, a result of an infection caused by group A beta-hemolytic streptococcus [18,25]. The incidence of RHD in North America had a significant reduction in the 20th century due to a decline in occurrences of rheumatic fever and the advent of antibiotics [25]. The RHD remains prevalent in underdeveloped nations, but there has been a global decline in its prevalence, while degenerative calcific MS is increasingly observed in the elderly population [18]. Furthermore, MR has emerged as the prevailing manifestation of MV conditions in industrialized societies [26]. It is linked to higher mortality rates and a higher incidence of heart failure diagnosis [25]. Regrettably, this condition is commonly not adequately treated, resulting in just 15% of patients ultimately receiving MV surgery [26]. According to the EuroHeart Survey, about one-third of the individuals had a native MV condition [18]. Specifically, 9.5% of patients were diagnosed with MS and 24.8% were diagnosed with MR. Among the 5001 patients included in the survey, MR was the second most prevalent diagnosis, while the survey observed a transition in the main cause of MR from RHD, which accounted for 14.2% of cases, to degenerative valve disease, which accounted for 61.3% of cases. Ischemic or secondary MR was found in 7.3% of cases [18]. Furthermore, this discovery was validated in a comprehensive national database in Sweden, where MR remained the second most prevalent VHD behind AS, with an occurrence rate of 21.3 per 100,000 person-years [27].

### 2.2. Transcatheter Mitral Valve Therapy

The introduction of MS treatment occurred in 1940 with the development of closed MV commissurotomy, which was characterized by Harken and Bailey as “finger-fracture valvuloplasty” [28]. In 1970, the introduction of cardiopulmonary bypass allowed for the switch from a closed surgical commissurotomy to an open one. Furthermore, in 1984, the Japanese cardiac surgeon Kanji Inoue presented the initial attempt for percutaneous therapy of VHD [29]. He and his colleagues presented five cases detailing the effective use of MV balloon valvuloplasty using venous access and transseptal puncture for the treatment of MS [29]. Retrograde non-transseptal balloon mitral valvuloplasty is a method of balloon valvuloplasty, developed by Stefanadis et al., with the aim of avoiding complications associated with transseptal catheterization for patients with symptomatic MS by using a dedicated steerable catheter; however, its use has declined over time [30].

Transcatheter MV balloon valvuloplasty is a procedure that resembles the closed surgical approach, and it involves the utilization of a balloon to rupture the MV commissures and restore mobility to the MV leaflets [31]. Randomized studies have demonstrated that compared to the surgical approach, it results in wider valve areas and improved permanence [31]. The initial utilization of this technique sparked curiosity in identifying anatomical factors that might forecast the effectiveness of a procedure through the use of echocardiography [3]. The outcome was the creation of the Wilkins score, which aims to assess the mobility, thickness, calcification, and sub-valvular thickening of the leaflets [32]. This score helps identify individuals with anatomies that are more likely to have successful procedures. This started the significant collaboration between imaging and intervention in the management of MV disease [32]. Currently, the most widely used approach for treating rheumatic MS is MV balloon valvuloplasty, which involves the use of the Inoue balloon trademarked by Toray Medical in Tokyo, Japan [33]. Kanji Inoue pioneered the development of structural VHD procedures, initiating a transformative shift and fostering new alliances that would restructure the treatment of MV conditions [25].

While percutaneous treatments for MS have been in existence and evolving for a considerable period, the development of procedural alternatives specifically for MR has only occurred in the last 20 years [25]. This progress has been made possible by scientific expansion, which has facilitated the creation of a growing array of percutaneous MV treatment options. Currently, there are a variety of equipment alternatives available that use distinct approaches and modes of operation. These devices can be deployed based on the specific underlying pathophysiology of the MV. The new advances may be broadly classified as processes that aim to target (I) leaflet repair; (II) direct or indirect annuloplasty; (III) implantation of artificial chordae; and (IV) transcatheter MV replacement [25]. Table 1 summarizes the currently available transcatheter treatments for MV repair.

### 2.3. Leaflet Repair/Edge-to-Edge Technique

**Figure 1:**
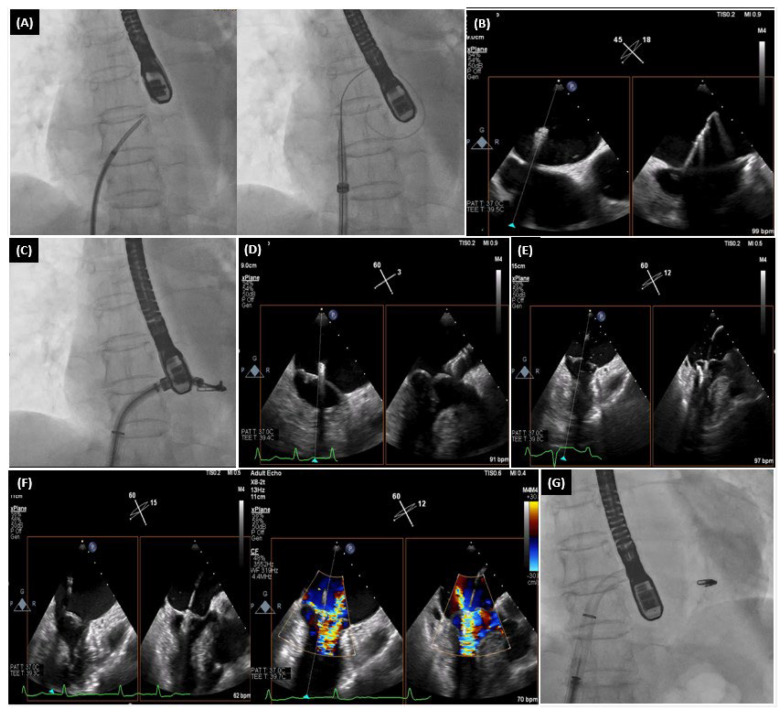
This is the case of a 71-year-old patient with hypertrophic cardiomyopathy, mitral valve prolapse with at least moderate MR, systolic anterior motion, and non-significant left ventricular outflow tract obstruction. This figure presents the MitraClip’s procedure steps: (**A**) transeptal puncture, (**B**) introduction of steerable guide catheter into the left atrium, (**C**) advancement of the clip delivery system into the left atrium, (**D**) steering and positioning of the MitraClip above the mitral valve, (**E**) advancing the MitraClip into the left ventricle, (**F**) grasping the leaflets and assessing for proper position, (**G**) release of the clip.

**MitraClip.** The MitraClip (Abbott Laboratories, Menlo Park, CA, USA) is constructed using cobalt chromium and is coated with a polypropylene tissue. The MitraClip device consists of two appendages and operates by bringing the margins of the anterior and posterior fragments of the MV leaflet closer together. This approach was developed based on the surgical “edge-to-edge” repair procedure, initially introduced by Alfieri et al. [34]. The MitraClip device obtained Conformité Européenne (CE) mark certification in Europe in 2008 and FDA clearance in the United States of America (USA) in 2013 for its application in primary MR [35]. Subsequently, in 2019, it was also approved for use in secondary MR. Currently, the MitraClip’s third generation is available commercially. This version includes two sizes: the original NTR size and the XTR size, which has clip appendages that are 3 mm lengthier [36]. The third generation of the MitraClip has enhanced steering, navigational, and positioning clip capabilities. These improvements make it easier to precisely place the clip and improve the accuracy of the procedure [36]. In order to attain a high eminence rate in reducing MR, it is important to address certain structural characteristics of the MV. The following principles were shown to be anatomical features that are advantageous for a proper device implantation in MR: a leaflet coaptation length greater than 2 mm, a coaptation depth less than 11 mm, a flail gap less than 10 mm in cases of primary MR, and a flail breadth less than 15 mm [35]. The increasing expertise in this procedure allows for the treatment of more intricate MV structures and alternations [35] (Figure 1).

As mentioned previously, transcatheter management of MR has lately become a significant option, particularly for individuals who have a high risk for surgery and suffer from functional MR. The initial transcatheter device offered was the transcatheter edge-to-edge repair (TEER) [15]. The frequency of TEER operations has notably escalated in recent years, with functional MR emerging as a primary indication for TEER. Following the completion of two significant studies, namely, MITRA-FR [37] and COAPT [38], a substantial debate ensued due to their contradictory outcomes. Specifically, MITRA-FR demonstrated no improvement in prognosis when compared to medical therapy [37]. Conversely, COAPT revealed that the MitraClip operation significantly reduced the mortality and hospitalization rates [38]. The disparities can be elucidated by the significantly elevated MR in the COAPT study. Additionally, the patients participating in the MITRA-FR study exhibited considerably worse left ventricular (LV) function, characterized by severe LV dilatation and dysfunction, in comparison to those in the COAPT trial [37,38]. Felbel et al. conducted a meta-analysis that compared the short-term and one-year results in individuals with functional MR who had TEER or surgical MV repair [39]. The researchers included 21 studies on TEER and 37 studies on surgery. They witnessed a noteworthy decrease in the in-hospital mortality with TEER, while there was no significant difference in the 1-year mortality [39]. Furthermore, one contemporary study conducted by Stone et al. has validated the previously observed low rates of short-term death associated with TEER [38]. Nevertheless, it has also been revealed that the long-term mortality among patients with decreased LV function and functional MR remains significant, with a 5-year mortality rate approaching 60% [38].

**PASCAL transcatheter mitral valve repair (TMVr) system.** The PASCAL percutaneous appliance, developed by Edwards Lifesciences in Irvine, CA, USA, obtained CE compliance in Germany in 2019. This device shares similarities with the MitraClip system, since both utilize the edge-to-edge repair procedure [34]. The purpose of this equipment was to address certain technical limitations of the MitraClip. It was designed with wider paddles and an interior spacer to make it easier to reduce MR and decrease tension and tethering on the MV. The unconstrained motion of the paddles allows for better grappling of the leaflets, particularly in difficult structures [40]. Additionally, the protraction of the equipment helps with its movement in the left ventricle (LV). The procedural processes are similar to those of the MitraClip [41]. The procedure involves initiation through the right femoral vein and utilizing the transseptal route to advance the PASCAL system into the LV using a 22 F guide catheter. The clinical data supporting the PASCAL implant remains weak. It was firstly implanted in 23 individuals as part of a conservative treatment in an observational study [41]. Excess MR of ≤2+ was reported in 96% of the participants and despite the presence of a bulky system, no significant rise in the trans-mitral gradient was identified. Furthermore, the CLASP trial assessed the safety and effectiveness of the PASCAL device in 62 individuals with moderate to severe mitral regurgitation [42]. After a period of 30 days, the major adverse events were 6.5%, while the overall mortality rate was 1.6%, with a residual MR of ≤2 in 98% of them. These encouraging findings need to be validated in a wider group of patients and with extended follow-up [42]. The ongoing CLASP II study will include a direct comparison between the well-established MitraClip device and the PASCAL device.

### 2.4. Indirect Annuloplasty Devices

**Carillon Mitral Contour System.** The Carillon Mitral Contour System (Cardiac Dimensions, Kirkland, WA, USA) is a medical device used for the treatment of patients with functional MR. It is a percutaneous indirect annuloplasty system, which comprises a self-expanding nitinol anchor at both the proximal and distal ends, which are joined by a shaping ribbon [43]. The Carillon System is utilized via the jugular vein using a 9F delivery catheter and it is inserted into the coronary sinus. By taking advantage of the close propinquity between the coronary sinus and the MV annulus apparatus, the diameter of the MV annulus will be decreased when the device is deployed, consequently reducing the functional MR [35]. This system has several benefits, including its user-friendly interface, unobtrusive construction, and the ability to easily retrieve and relocate the system if necessary. The earliest trials assessing this device demonstrated an amelioration of symptomatology, a decrease in MR, and an enhancement in LV remodeling metrics [43]. Regarding the Carillon system, due to its outline and precise placement, experiencing significant tension at the proximal anchor, asymptomatic fractures of the device were seen in 25% of patients in this area. Hence, a revised apparatus was created and assessed in the prospective single-arm safety TITAN II study [44]. The enhanced clinical and echocardiographic outcomes observed in prior investigations were validated in the TITAN II study [44]. There was a solitary instance of device fracture, accounting for 2.8% of all cases. The primary endpoint, major adverse events at 30 days, also occurred in 2.8% of the patients. The mortality rate after twelve months was 23%, and none of the fatalities were attributed to the device [44]. In July 2019, the Carillon System was successfully implanted in its 1000th case, which serves as evidence of its widespread use in the field of transcatheter MV replacement. A double-blind randomized study, the REDUCE-FMR trial, was conducted to objectively assess the effectiveness of this system [45]. In this study, 87 symptomatic patients with functional mitral regurgitation under optimal medical treatment were randomized to either receive this treatment or sham and a decrease in MR and LV remodeling was demonstrated in patients treated with the Carillon System [45].

### 2.5. Direct Annuloplasty Devices

**Cardioband system.** The Cardioband device (Edwards Lifesciences, Irvine, CA, USA) is a minimally invasive system used for the treatment of functional MR. It functions similarly to annuloplasty, a surgical technique to repair the MV and improve its performance. The device is built based on the surgical annuloplasty devices; however, it is administered using a percutaneous trans-septal technique. The Cardioband is a flexible band that is surgically inserted from the anterolateral to the posteromedial commissure, using many tiny anchors at the structure [46]. The procedure is conducted with the assistance of continuous echocardiographic and fluoroscopic assistance. Once the band is securely attached to the annulus, the implant is used to decrease the size of the MV annulus, improve the alignment of the leaflets, and thus decrease MR [35]. A multicenter trial including 60 patients who received the Cardioband system showed satisfactory outcomes after one year [46]. The 1-year survival rate was 87% and moderate or less residual MR was found in 61% of the cases. Alongside, there was a considerable improvement in functional status, quality of life, and exercise ability. In the initial stage of the trial, this device encountered technical issues, where 9 out of 10 patients had anchor disengagement, resulting in an ineffective outcome for 5 patients, and as a result, it underwent modifications throughout the initial phase of the investigation [46].

**Mitralign system.** The Mitralign annuloplasty system (Mitralign, Tewksbury, MA, USA) is based on the circumvolution of the posterior annulus using a device that delivers pledgets. This device is inserted through the aorta in a retrograde manner, allowing for access to both the LV and left atrium (LA). Pledgets are positioned in sets at the antithetical ends of the annulus to decrease the diameter of the annulus, which leads to a depletion in MR [35]. The Mitralign system obtained CE mark clearance in 2016 based on the safety and effectiveness results presented in the CE mark study. The data from the prospective, multicenter, single-arm trial showed a notable enhancement in the 6 min walk test and a refinement in the size and remodeling of the LV after 6 months. MR depletion was observed in 50% of the cases, with a device success rate of 70.4%, while tamponade occurred in 8.9% of the patients, although no mortality events occurred [47].

**Memo 3D ReChord ring.** The highly stiff circular ring Memo 3D ReChord, developed in United Kingdom, is equipped with a chordal guiding system. Its purpose is to facilitate the implantation of artificial neochordae, ensuring precise and successful results without the requirement for specialized chordal measurement. There are limited data on this technique, with a Greek series of 10 patients with promising initial results [48].

### 2.6. Chordal Replacement

Chordal replacement, a frequently used procedure for patients in need of cardiac operations, is yet to establish itself as a minimally invasive treatment rather than a transcatheter therapy. Being an MV treatment, it conceptually addresses the pathology of MR mainly using the transapical approach. Currently, there are two systems being utilized, namely the NeoChord DS1000 and the TSD-5 MV repair device [35].

**The NeoChord DS1000 device.** The NeoChord device (NeoChord Inc., St. Louis Park, MN, USA) is a surgical technique used to overhaul the MV in patients suffering from degenerative MR without the need for a heart–lung machine (cardiopulmonary bypass). The surgery is conducted with the use of general anesthesia, utilizing the transapical access to insert artificial chords into the MV leaflets [35]. Once the neochordae are secured within the specific leaflet region, the chordae will be fastened at the entry point of the LV. The outcomes of the FIH TACT study resulted in the device receiving CE mark certification in 2012. Each of the 30 participants in the study experienced a prolapse of the posterior MV leaflet accompanied by either chordal rupture or elongation and repeat procedure necessitated in six individuals [49]. Since 2012, NeoChord devices have been utilized in over 450 cases and their data are all collected in a proceeding registry. Furthermore, a crucial study conducted by the FDA, known as the RECHORD study, has commenced with the objective of recruiting 585 patients across 20 engaged centers.

**TSD-5 mitral valve repair device.** The TSD-5 MV repair system (Edwards Lifesciences, Irvine, CA, USA) is a homogeneous appliance to the aforementioned NeoChord, since it also utilizes the transapical approach. The manufactured cords are inserted accompanied by transesophageal echocardiography and fluoroscopic employ at the posterior MV leaflet using their specialized equipment. Once the proper length of the chordae has been confirmed, they are secured outside the LV using Teflon substance [35]. The initial findings were presented at the Transcatheter Cardiovascular Therapeutics Conference in 2016, demonstrating a procedural success rate of 100%. However, there were two instances of repeat procedures caused by pericardial effusions and two more due to persisted MR during the follow-up [50]. A CE mark study is now being conducted at 22 centers in five countries across the European Union.

### 2.7. Transcatheter Mitral Valve Replacement (TMVR)

The intricate structure of the MV, characterized by its semi-circular shaped, oblique annulus, complicated sub-valvular apparatus, and vast and wavering acreage, presents significant challenges for mitral valve replacement (MVR). The primary obstacles in the creation of proper TMVR prostheses involve securely attaching them to a semi-circular shaped, native MV without calcific deposits to prevent the prosthesis from slipping into the LV [35]. Additionally, it is important to avoid substantial paravalvular leak (PVL) and obstruction of the LV outflow tract (LVOT) by supplanting the anterior MV leaflet into the LVOT. Several prostheses outlines have been developed to tackle these obstacles, with some of them already being tested in individuals as part of initial-phase trials [51].

**Access site.** There are two approaches available for performing TMVR: the transfemoral (TF) venous approach with transseptal (TS) puncture, and the transapical (TA) approach of the LV [51]. The first route is considered a vaguely invasive method, especially compared to the second one, and it allows for an antegrade access to the MV. However, it might present certain difficulties due to its methodology. On the other hand, anterolateral thoracotomy is necessary for the transapical approach. This method is more invasive; nevertheless, it is a commonly performed procedure with the least possible invasiveness. The operator uses this technique to directly approach the MV, and this permits the accurate placement, securing, and insertion of any TMVR bioprosthetic valve [52].

**Prostheses.** Various bioprosthetic valves are accessible, each utilizing distinct techniques, anchoring, and sealing methods. The global exposure to various valves remains constrained. Every prosthesis is composed of a self-expanding frame, often made of nitinol, which includes leaflets, usually from bovine or pig pericardium, to create the bioprosthesis. The available THVs that are being tested are mentioned below (Table 2) [53]:

1. The Intrepid TMVR system, manufactured by Medtronic in Minnesota, features a flexible dual-stent architecture that operates without the necessity for alternating alignment. The results of the Intrepid TMVR Early Feasibility Study [54] indicate that the advantages of this THV were sustained for up to 12 months, with low mortality, low need for re-operation, and almost outright removal of the MR. These findings demonstrate a favorable safety profile and long-lasting functionality of the valve.
2. A completely recoverable and re-adjustable TMVR system is the Tendyne, made by Abbott Vascular in California. A 34 F sheath is utilized for its implantation through the apex. It features a porcine pericardial valve with three leaflets encased in two self-expanding stents from nitinol. The Real-World Tendyne European Experience Registry [55] outcomes indicate an elevated probability of technical success, long-lasting and thorough elimination of MR, notable therapeutic advantages, and a cardiovascular mortality rate of 17% within 12 months following the TMVR.
3. The Tiara, developed by NeoVasc in Canada, is a device that includes bovine pericardial leaflets, and it is embedded within a self-expanding nitinol compound.
4. The AltaValve, developed by 4C Medical in Minnesota, is a distinctive THV that is positioned in the LA. The device is composed of a nitinol body that is self-expanding with an elongated form and inside this, there is a bovine pericardial valve.
5. The Cardiovalve, developed in Israel, is a self-expanding THV made from bovine pericardium. It consists of two segments, one for the LA and one for the LV. The product is offered in three different shapes, and it is provided using a 28 F sheath.
6. The Cephea system, developed by Abbott Vascular, has a self-expanding structure with two plates. The inner compound contains a valve from bovine pericardium, and its layout enables the outer disk to adapt to different shapes while segregating the rest THV.
7. The EVOQUE valve, developed by Edwards Lifesciences in California, is a self-expanding THV made of nitinol, with leaflets derived from bovine pericardium. The LV skeleton consists of nine hooks that connect to the leaflets of the MV and chords. The LA component of the device offers extra support by attaching to the annulus and it includes a skirt that prevents PVL. The initial trial conducted in the USA [56] has shown that the transseptal TMVR system is possible, with technical success in 92.9% of the patients, while non-cardiovascular death occurred in 7.1% of cases. The system resulted in improved MR and the overall clinical state of the patients.
8. The HighLife system, developed by HighLife Medical in France, utilizes a sub-annular ring that is attached over the MV via a TF approach. This structure functions as a stabilizing point for the self-expanding THV, which incorporates three leaflets from bovine pericardium, and it can be implanted either via the apex of the heart or through the interatrial septum.
9. The SAPIEN M3 system (Edwards Lifesciences, Irvine, CA, USA) consists of a nitinol structure that surrounds the MV and inside is attached a balloon-expandable THV through the interatrial septum. The valve resembles the SAPIEN 3 THV; nevertheless, it features an extra skirt to assist in sealing, avoiding any PVL. The ongoing ENCIRCLE trial (ClinicalTrials.gov identifier: NCT04153292) is designed to evaluate the safety and efficacy of the SAPIEN M3 THV in individuals with symptomatic, severe MR who are not suitable candidates for surgery or other percutaneous procedures.

### 2.8. Mitral Valve-in-Valve (MVIV), Valve-in-MAC (TMVR in-MAC), and Valve-in-Ring (MVIR)

Mitral valve replacement and repair have been the mainstay of the MV surgical treatment in recent years [53]. Opting for MV repair is more advantageous than MVR, since it has demonstrated superior prognostic outcomes and, also, delays the necessity for MVR in comparatively younger individuals. This is crucial, as we now lack an optimal alternative for MVR, even though younger individuals are often recommended to have mechanical valves, which have a longer lifespan but necessitate anticoagulation [57]. Conversely, older patients are advised to have a bioprosthetic valve (either pericardial or porcine) to avoid the requirement for anticoagulation. These valves degenerate over time, and their lifespan in the MV ranges from 10 to 15 years [58]. Prior to recent advancements, repeat procedure was the sole recourse for these cases. However, the success of TAVI and valve-in-valve (VIV) procedures for damaged aortic bioprosthetic valves has introduced a new option known as MV valve-in-valve (MVIV) [59,60]. In this procedure, a TAVI THV in implanted within the failing valve in the mitral position. Although MVIV shares many similarities with other procedures, it possesses distinct characteristics in terms of case screening, operation preparation, post-operative care and possible complications [61]. Annuloplasty is a crucial component of MV repair and is executed to restructure and strengthen the MV annulus. This is accomplished by applying an annuloplasty ring or band to the atrial side of the MV annulus. Like MVIV, repair deficits have been addressed by inserting a TAVI THV within the ring, known as MV valve-in-ring (MVIR) [59]. Furthermore, mitral annular calcification (MAC) is a gradual and degenerative process in which the MV becomes calcified and is commonly linked to MS, MR, or a combination of the two. Individuals diagnosed with MAC have limited suitability for surgical treatment due to the complex technical difficulties involved and the elevated risk of death during the peri-operative period. TMVR has become a viable choice for those individuals and is elucidated by the utilization of the transcatheter valve-in-MAC procedure [35]. Precise anatomical evaluation is crucial in order to prevent adverse events associated with TMVR, including LVOT obstruction, THV migration, embolization, and PVL [58].

### 2.9. Critical Appraisal of Transcatheter Mitral Valve Interventions

Although currently available percutaneous MV procedures are limited to patients who are deemed inoperable or have a very high surgical risk, such patients are frequently encountered in clinical practice. Therefore, these therapies are continuously evolving and will become a mainstream therapy over the following years. Currently, devices for TEER are by far the most common transcatheter treatment, especially for patients with secondary mitral regurgitation. Despite the limited clinical experience, the newer transcatheter repair options may provide an alternative or complementary option in patients who are not anatomically ideal candidates for TEER, while developments in TMVR, especially with an eventual decrease in the profile of delivery systems, may provide a more comprehensive option for patients with MR.

## 3. Transcatheter Interventions for Tricuspid Valve Disease

Due to the unsatisfactory results of performing surgery solely on TV, researchers are currently examining various transcatheter treatments for it. The percutaneous techniques may be classified into four main categories: edge-to-edge repair, TV replacement (TVR), TV annuloplasty, and palliative TV treatment [20].

### 3.1. Transcatheter Edge-to-Edge Repair (TEER) of the Tricuspid Valve

**TriClip.** The TriClip system (Abbott Cardiovascular, Plymouth, MN, USA) is a TEER device that demonstrates high efficacy in mitigating the severity of TR [62]. The system utilizes the surgical technique of edge-to-edge valve approach using a 24 Fr TF delivery device. The system comprises a 4 mm cobalt–chromium clip and two gripper arms that can be operated separately, allowing for unrestrained opening and closing. The clip is transported via a controllable catheter, and it secures itself in position by grasping the TV leaflets. After the MitraClip proved to be successful, it was used for the first time in 2015 on three patients with severe TR. The procedure was successful in all cases, with no mortality events occurring during the procedure, demonstrating its feasibility [63].

The MitraClip and TriClip platforms comprise a navigable guiding catheter and a clip delivery structure. The components of this system consist of a controllable sleeve, a catheter for delivery, and a clip with two movable arms, measuring either 4 mm (NT size) or 7 mm (XTR size) in width [62]. The clip is designed to securely hold and pull the TV leaflets. The MitraClip XTR the platform’s 3 mm longer clips are beneficial for individuals who have bigger coaptation gaps. The clip delivery structures are advanced using the conduit given by the guiding catheter to modify the implanted clip. Unlike the conventional MitraClip system, the new TriClip design includes two knobs specifically designed for steering maneuvers [64]. Furthermore, the steerable sleeve possesses a solitary knob for tip deflection, and its distal curvature exhibits a reduced radius. The different versions of the TriClip system enhance the ability to perform bending and directing maneuvers within the right atrium (RA) [64].

The TV TEER has now become the most often conducted percutaneous procedure for TV globally. In 2019, the Transcatheter Tricuspid Valve Therapies (TriValve) registry published the 1-year results of 249 individuals who had attempted TEER with the TriClip [65,66]. Procedural success was observed in 77% of the cases, with each patient receiving 1–3 clips. At the one-year mark, 72% of patients showed a decrease in TR and 69% showed a refinement in their New York Heart Association (NYHA) functional class, with a level of II or below [66].

The TRILUMINATE single-arm trial subsequently evaluated the safety and effectiveness of the TriClip technology [67,68]. Data were provided for the outcomes at 6 months and 1 year. After 6 months, 86% of the cases had a decrease in the intensity of TR by at least one grade, while 4% of them died during that period. After 1 year, 71% of the cases continued to see a decrease in TR severity, while 83% of them showed refinements in NYHA functional class and their all-cause mortality rate was 7% [67].

The TRILUMINATE Pivotal study (ClinicalTrials.gov identifier: NCT03904147) aimed to compare patients who are randomly assigned to receive medical therapy with those who receive TriClip [69]. For that reason, 350 patients were randomized, and the results showed significant improvement in quality of life, related to accountable TR reduction, while there were no differences regarding mortality and heart failure hospitalization [69]. TriClip technology has achieved a high level of technical success, and operators are quite experienced with it. As a result, TV TEER has become the benchmark for repairs performed by percutaneous procedures.

**PASCAL transcatheter valve repair system.** The PASCAL Repair System (Edwards Lifesciences, Irvine, CA, USA) comprises an extension and two clasps that firmly hold onto the TV [41]. The clasps may be handled separately and adjusted until the ultimate equipment is delivered, enabling the gradual leaflet apprehension. The device is inserted using a 22 Fr TF method and was initially developed for the treatment of MR [41,70]. In 2018 [71], the first instance of TV implantation utilizing the PASCAL Repair System was documented in a patient suffering from severe/torrential TR. After a 30-day period, the patient’s NYHA class decreased from IV to II, there was a refinement in the 6 min walk test, and an enhanced quality-of-life score. The transthoracic echocardiogram during the follow-up examination showed a decrease in TR to a moderate level. Following the initial case presentation of the PASCAL Repair System for TR, a paper was published detailing the first-in-human compassionate use experience including 28 individuals [72]. The procedural success rate was 86%, with no procedural mortality events. During the 30-day follow-up period, two patients passed away and one patient’s death was attributed to a probable cardiac etiology, while the other patient died owing to a rehospitalization for heart failure. The findings of the one-year follow-up showed a survival rate of 93% and 86% of patients had sustained moderate or less TR. Approximately 90% of the cases exhibited NYHA functional class I or II and shown improvement in the 6 min walk test. The CLASP TR early feasibility trial (Edwards CLASP TR EFS; ClinicalTrials.gov identifier: NCT03745313) released the 30-day adverse events in 2021 [73]. The trial had a total of 34 individuals, out of whom 29 underwent implantation of the PASCAL Repair System. After 30 days, there were no deaths related to cardiovascular issues and 85% of the cases saw a decrease in TR of at least one grade, while 70% experienced a decrease of at least two grades [73].

### 3.2. Tricuspid Valve Replacement

Table 3 summarizes the transcatheter valves that are currently available for implantation in the tricuspid position.

**EVOQUE tricuspid valve replacement system.** The EVOQUE system (Edwards Lifesciences, Irvine, CA, USA) is a bioprosthetic THV made of bovine tissue attached to a self-expanding metal (nickel–titanium) frame for support [74]. It is administered using a 28 Fr TF system and is then secured within the TV [62]. The EVOQUE valve is available in several sizes (52 mm, 48 mm, and 44 mm) to effectively treat a wide spectrum of TV conditions and anatomical alternations. The initial case was documented in 2020 by Fam et al. in a solitary individual, with procedural success, and after 6 months, minimal residual TR, NYHA class I symptomatology, and convalescent quality-of-life [75]. The TRISCEND trial, conducted at many centers, examined the safety and efficacy of this valve in a total of 132 patients [76]. By the 6-month mark, all 43 cases who were present for follow-up witnessed a decrease in TR to mild or below and 89% of them showed an improvement in their NYHA class, reaching class I or II and enhancement in their overall quality of life. The initial success of the EVOQUE valve has led to the initiation of the TRISCEND II study (ClinicalTrials.gov identifier: NCT04482062), which randomized more than 700 individuals to either the EVOQUE valve or optimal medical care [77]. The initial results of this trial were presented in the Transcatheter Cardiovascular Therapeutics (TCT) congress in San Francisco in 2023. After 30 days, the primary composite safety endpoint was observed in 27.4% of the 95 cases, lower than the historical safety data following TV surgery (43.8%). The two most often observed adverse events were severe bleeding (10.5%) and requirement for permanent pacing (14.7%), while the rate of cardiovascular death was 3.2%. At 6 months, EVOQUE valve implantation significantly reduced TR, with 98.8% of the individuals having moderate TR or lower, and 78% of the cases no or negligible TR. Ultimately, the enhancements in both quality of life and functional status were much better with EVOQUE, demonstrated by a win ratio of 4.6 [77].

**GATE system.** The GATE system (NaviGate Cardiac Structures, Lake Forest, CA, USA) is a self-expanding THV made of nitinol and is covered with pericardial membrane. It is shaped somewhat like a truncated cone, and it has 12 winglets and 12 graspers to securely attach the device in the TV. The THV can be deployed either by a 42 Fr internal jugular approach or transatrially with a mini thoracotomy [62]. The stent comes in five sizes (36, 40, 44, 48, and 52 mm) to meet various anatomical contingencies and in 2017 it was tested in two patients, both of whom had a successful procedure [78]. Subsequently, 34 patients received the valve, with effective delivery in 85% of them. The procedure was performed using a transatrial route in 25 patients and a transjugular (TJ) approach in 4 individuals. At 30 days follow-up, 17% of the cases needed conversion to surgery, and 24% died in the transatrial group and 33% died in the transjugular group. Due of the unexpectedly high mortality rate, researchers are presently studying a bigger population over a longer period of time [62].

**INTREPID system.** The INTREPID system (Medtronic, Fridley, MN, USA) is an innovative biological, self-expanding stent THV made of nitinol and bovine membrane. It may be inserted through a 35 Fr venous approach, and it is offered in three different exterior frame sizes (43 mm, 46 mm, or 50 mm), accompanied by an interior stent frame of 27 mm. Its main advantage is that it does not rely on leaflet capture for anchoring [62]. Instead, it is secured by deploying a wide, atrial brim on the atrial side of the THV. A study conducted between 2015 and 2017 involved 50 patients with procedural success in 98% with the THV in the MV position. In 2020, a small study documented the use of the device in the TV in three patients, with effective delivery in all of them. A preliminary study is now being conducted in the USA to assess the viability of employing the system for treating TV conditions (ClinicalTrials.gov identifier number: NCT04433065) [19].

**LuX-Valve.** The LuX-Valve (Jenscare Biotechnology, Zhejiang, China) is another self-expanding, nitinol THV using bovine tissue to form the three leaflets. The valve is equipped with an atrial disc and a septal anchor, which has two graspers for securely attaching the leaflets [62]. The delivery may be performed using a 32 Fr catheter through a small-incision thoracotomy. The device is offered in three sizes of discs, 50 mm, 60 mm, and 70 mm, and two sizes of THVs, 26 mm and 28 mm. The initial use in human subjects was documented in 2020 and encompassed a cohort of 12 individuals [79]. All patients had procedural success; however, there was one mortality event within 30 days after the procedure. Another study, conducted in 35 patients, demonstrated a procedural success rate of 100% and a 30-day mortality rate of 6%. It is worth mentioning that, like the INTREPID system, the LuX-Valve has been effectively inserted into a minimum of five patients who already had pacemaker leads, without causing any interruption [62].

**TricValve—Transcatheter Bicaval Valve System.** The TricValve is a TF prosthesis with two self-expanding caval THVs, manufactured in Austria. This THV is designed with an inner bulge to enhance its stability and avoid displacement. Furthermore, it has a skirt to minimize any PVL, and an exposed upper section to provide unobstructed circulation through the vein. The inferior vena cava (IVC) THV has a better radial force to provide stability and a skirt to prevent occlusion of the hepatic vein. THV implantation needs to be initiated at a high position to enhance stability and enable re-adjustment of up to 80% of the implantation. During the installation of the IVC THV, it is important to note that the THV includes a piece that is covered, and its upper part needs to be positioned close to the junction of the RA. Superior vena cava (SVC) sealing is feasible for individuals with a cardiovascular implantable electronic device (CIED) who have leads that are overridden by the THV. This procedure circumvents classic structural limitations of the TV procedures and can be conducted without the need for general anesthesia. The TRICUS EURO trial, which examined 35 patients, reported a procedural success rate of 94% [80]. However, one case of THV migration occurred, but no embolization was observed. At the six-month follow-up, there were no notable disparities in echocardiographic data, with a substantial reduction in loop diuretic dose, Kansas City Cardiomyopathy Questionnaire (KCCQ) score, and NYHA class [80].

### 3.3. Tricuspid Valve-in-Valve and Valve-in-Ring

The durability of surgical TV prosthesis is inferior to that of other prosthetic valves [81,82]. The decision to utilize mechanical prostheses in the TV when repair is not possible is less frequent compared to left-sided valve treatment approaches, mostly due to the increased likelihood of prostheses thrombosis in the low-pressure environment [83]. Furthermore, the utilization of the bioprosthetic valves might potentially increase the incidence of prostheses failure, characterized by either regurgitation or stenosis. In many patients with many comorbidities, a redo surgical procedure is generally impractical. As a result, the potential for transcatheter valve-in-valve (TVIV) and transcatheter valve-in-ring (TVIR) procedures becomes an appealing choice [62].

The TVIV and TVIR techniques have been thoroughly explained in the past [84,85]. The usual access approach is the TF; however, in certain situations with horizontally positioned valves, access through the jugular vein may be employed. Presently, there are two THVs that are available for utilization: the Melody THV manufactured by Medtronic in Minneapolis, Minnesota, and the SAPIEN 3 THV manufactured by Edwards Lifesciences in Irvine, CA, USA. Furthermore, TVIR is a viable option for patients who already have pacemaker leads, and the process of jailing the device lead is often safe and well-tolerated. Nevertheless, if the patient is pacing-dependent, it is crucial to closely monitor the lead impedance and thresholds right after the device is implanted to guarantee optimal performance [84,85]. An important consideration regarding the implantation of a transcatheter valve in a tricuspid valve ring, is that open rings are mostly used in this surgery, which do not provide full support for transcatheter valves, thus limiting the number of cases that can be treated by this approach.

**Figure 2:**
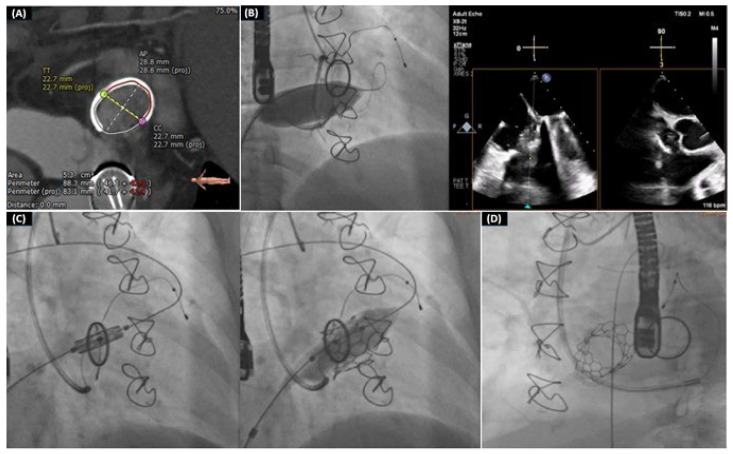
This is the case of a 53-year-old female patient with progressive dyspnea (NYHA III), past medical history of breast cancer (mastectomy, chemotherapy, and radiation therapy), surgical MV management with mechanical prosthesis (St Jude 23 mm), and tricuspid valve repair with annuloplasty ring (Edwards physio ring 28 mm). This figure presents the valve-in-ring’s procedure steps: (**A**) pre-procedural screening with analysis of the cardiac computed tomography, (**B**) balloon testing for right coronary artery occlusion, (**C**) transcatheter heart valve implantation, (**D**) final result.

In 2011 was the first successful TVIV utilizing a 23 mm SAPIEN valve and then subsequent case reports and registries have further substantiated the efficacy of this treatment option [86,87]. The TVIV registry reported a procedural success rate of 99%, among whom 62% were implanted a Melody THV, while the other patients received a SAPIEN THV. After a median follow-up period of around 400 days, 14% of the cases died, with five of them dying within 30 days. Crucially, the results were not influenced by the type of THV that was inserted [88] (Figure 2).

### 3.4. Critical Appraisal of Transcatheter Tricuspid Valve Therapies

Severe TV conditions have significant adverse outcomes and have unclear guidelines for their treatment options, resulting in a high risk of death after surgery. Considering the hesitancy in performing isolated TV surgery, especially after left-sided heart valve surgery, transcatheter treatment appeals as a promising alternative for managing the increased morbidity arising from this condition. Interventionalists now have more percutaneous choices for TV pathologies, with TEER devices accumulating evidence that facilitates mainstream adaptation. Although more data are available for these devices, transcatheter TVR arises as a promising alternative for the future; however, outcome data are currently scarce and additional evaluation is warranted.

## 4. Transcatheter Pulmonary Valve Therapies

The Melody THV, developed by Medtronic Inc. in the United States, initially appeared by deploying a novel 18 mm THV on a 12-year-old child by Bonhoeffer et al. [23]. This THV consisted of a bovine jugular vein across a platinum stent. This procedure was performed on a 12-year-old child by Bonhoeffer and colleagues. The latest version of this THV consists of the same tissue that has been embroidered into a stent from platinum. It is accessible in two sizes (20 and 22 mm) and it is implanted via the Medtronic Ensemble device, which employs a “balloon-in-balloon” mechanism [22].

Prior to its utilization in the USA, European centers already had extensive competence with the Melody THV. Lurz et al. presented a study on 155 patients, achieving a remarkable success rate of 96.7% [89]. They observed an absence from the need for repeat operation of 70% within ~6 years, and noted they further decreased at experienced centers [89]. In 2015, the United States Investigational Device Exemption trial released data on the medium-to-long-term outcomes [90]. They included 171 patients, and 148 of them successfully received THVs. The absence of re-operation cases after 60 months was 76%. The Melody THV obtained a CE mark certification in 2006, while the FDA awarded its full approval in 2015 [91].

The Edwards Sapien XT THV, developed in USA, received FDA authorization for its use in the PV in 2016. The THV is composed of bovine pericardium attached to a cobalt chromium stent. The stent is equipped with a skirt on its bottom exterior section to reduce PVL [22]. The COMPASSION study included a total of 81 individuals, out of which 69 received this THV. The study found that these patients had a 93.7% rate of not requiring further intervention within a period of 36 months. Furthermore, there are studies being conducted to assess the effectiveness of the Edwards Sapien 3 THV [22]. This THV offers an alternative of a 20 mm size and is considered more suitable for delivering the THV into the RVOT. An analysis of a cohort of 56 patients in Germany indicates that the procedural success rate was great, and there were no instances where further procedures were required over the 24 months follow-up period [92].

The Venus *p* valve, manufactured in China, is an innovative THV that incorporates leaflets from porcine pericardium within a self-expanding nitinol stent. The central section has dimensions ranging from 22 to 36 mm and an outline ranging from 20 to 35 mm. The central and distal ends are expanded to create a cylindrical structure, which aids in securing the device in the RVOT and the major pulmonary vessels [93].

The Pulsta valve, developed in South Korea, is a distinct THV device that has a comparable principle. The device is equipped with a self-expanding double-strand nitinol body, onto which porcine pericardium leaflets are attached. The THV is offered in diameters ranging from 18 to 28 mm, with intervals of 2 mm. The stent structure has a length of 28–38 mm, which is contingent on the dimensions of the THV. The ends of the structure are flared outwards by an additional 4 mm compared to the outside diameter in order to provide attachment [94].

## 5. Special Considerations—Valve-in-Valve Procedures

The implantation of a THV inside a bioprosthetic valve often involves a less intricate process. The danger of coronary constriction is reduced due to the inability to excessively expand the THV and typically pre-stenting is unnecessary. The category of the bioprosthetic device is essential as its inner diameter directly determines the size of the THV. There are several presentations of valve fracture, which involve using a high-pressure balloon to expand the prothesis to a width equal to or slightly greater than its outer diameter [9]. This prevents the occurrence of patient prosthesis mismatch (PPM) in the future. Fracture can be safely conducted in the PV before the deployment of the THV. It is important to evaluate the structure of the coronary arteries while evaluating the possibility for prosthesis fracture [59,62].

## 6. Conclusions

Currently, percutaneous valve treatment is a well-established therapy option for individuals with MR and rheumatic MS who are at a high risk for surgery. The most recent demonstration of the advantages of TEER in selected patients has shown its effectiveness in treating secondary MR. Nevertheless, the efficacy of percutaneous repair and replacement for primary MR has not yet been revealed. However, additional research is required to determine if mitral repair and replacement should be rejected or utilized in conjunction, particularly in relation to the compatibility of the prosthesis with the anatomy of every person. The adaptation of an implant is accompanied by significant economical limitations. Consequently, in the future, individuals with uncommon anatomical features may still only have the option of undergoing open surgery.

The clinical implementation of percutaneous TV procedures is still in its early stages. The progress of the research has probably been hindered by the absence of acknowledged influence of TR on the clinical presentation and the prognosis. Nevertheless, it now serves as an appealing option for individuals who have isolated secondary TR, as a second option to a potentially hazardous surgical procedure. However, all the necessary stages in creation of a novel THV design still need to be completed for percutaneous TV procedures.

Percutaneous PV replacement has had significant advancements in the last ten years and is now a widely used procedure for treating patients with impaired RVOT function. The short- and midterm outcomes are generally favorable, with a low and tolerable incidence of morbidity and mortality. In an ideal scenario, further advancements will lead to the development of more compact and adaptable delivery methods. Given the recent implantation of the present THVs and the limited follow-up, it is imperative to collect future data in order to ascertain the overall durability of these devices and the specific forms of valve failure that may occur. In addition, as many patients have narrower and obstructed passages, even in a developing environment, implementing novel surgical methods to enable greater growth of these tiny and obstructed passages might have substantial therapeutic advantages.

Considering the intricate nature of MV, TV, and PV conditions, as well as the growing range of therapeutic options available, it is crucial for the Heart Team to engage in the discussion to ensure that each patient is provided with a suitable treatment based on current scientific data.

## Figures and Tables

**Table 1:** Major devices for transcatheter mitral valve procedures.

Category	Device		Narrative	Access	Certification/Approval
TEER	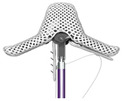	MitraClip (Abbot Vascular, Chicago, IL, USA)	“Edge-to-edge” technique by Alfieri et al. [34]	TF-TS	>80,000 implantations, CE and FDA approval
	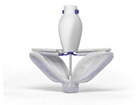	PASCAL (Edwards Lifesciences, Irvine, CA, USA)	“Edge-to-edge” technique by Alfieri et al. [34]	TF-TS	CE approval
Direct Annuloplasty	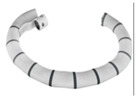	Cardioband (Edwards Lifesciences, Irvine, CA, USA)	Adjustable band to the posterior annulus	TF-TS	CE approval
	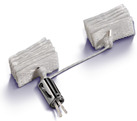	Mitralign (Mitralign, Inc., Tewksbury, MA, USA)	Enlargement of the posterior annulus with two pairs of pledges	TF-TS	CE approval
	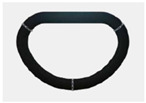	Memo 3D ReChord ring	Implantation of artificial neo-chordae	TA	CE approval
IndirectAnnuloplasty	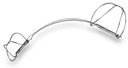	Carillon (Cardiac Dimensions, Lake Forest, CA, USA)	Nitinol anchors placed in the distal and proximal coronary sinus	TJ	CE approval
ChordalReplacement	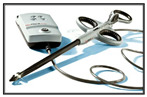	NeoChord (NeoChord, St. Louis Park, MN, USA)	Implantation of artificial chords	TA	CE approval
	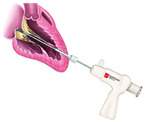	TSD-5 device (Harpoon Medical, Inc., Baltimore, MD, USA)	Implantation of artificial chords	TA	Ongoing trial for CE approval

TEER: transcatheter edge-to-edge repair; TF: trans-femoral; TS: trans-septal; TJ: transjugular; TA: transapical; CE: conformité européenne; FDA: Food and Drug Administration; IL: Illinois; CA: California; MA: Massachusetts; USA: United States of America; MD: Maryland; MN: Minnesota; Inc.: incorporation.

**Table 2:** Summary of available transcatheter mitral valve replacement bioprosthesis.

Device	Frame		Leaflets	Anchoring	Delivery	Recapturable
Intrepid	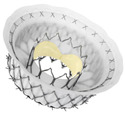	Dual-stent self-expanding/nitinol	Bovine PD	Perimeter oversizing	TS	Yes
Tendyne	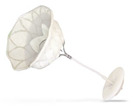	Dual-stent self-expanding/nitinol	Bovine PD	Apical pad	TA	Yes
Tiara	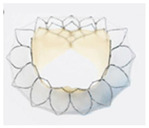	Self-expanding/nitinol	Bovine PD	D-shaped configuration with three anchors	TA	No
AltaValve	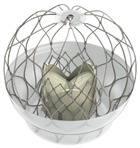	Spherical self-expanding/nitinol	Bovine PD	Supra-annular valveanchored in left atrium	TA/TS	Partially
Cardiovalve	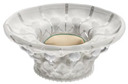	Dual-frame self-expanding/nitinol	Bovine PD	MV leaflets/annulus	TS	Partially
Cephea	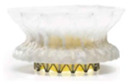	Dual-stent self-expanding/nitinol	NA	MV annulus: double disk	TS	Yes
EVOQUE	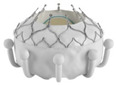	Self-expanding/nitinol	Bovine PD	MV leaflets/annulus	TS	No
HighLife	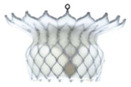	Self-expanding/nitinol	Bovine PD	Sub-annular ring (valve-in-ring)	TS	No
SAPIEN M3	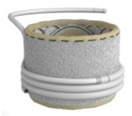	Balloon-expandable cobalt–chromium	Bovine PD	Sub-annular nitinol dock	TS	Partially (dock)

PD: pericardium; NA: not applicable; TS: trans-septal; TA: transapical; MV: mitral valve.

**Table 3:** Summary of available transcatheter tricuspid valve replacement bioprosthesis.

Device	Frame		Leaflets	Anchoring	Delivery
EVOQUE	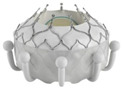	Self-expanding (nickel–titanium)	Bovine PD	Intra-annular sealing skirt and anchors	TF
GATE	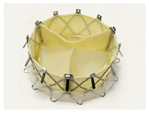	Self-expanding/nitinol	Xenogenic PD	Atrial winglets, ventricular graspers	TJ
INTREPID	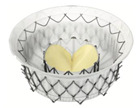	Dual-stent system	Bovine PD	Recoverable before release	TF
LuX-Valve	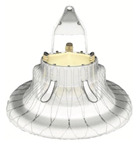	Self-expanding/nitinol	Bovine PD	Leaflet fixation/septal anchoring	TA
TricValve	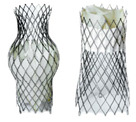	Self-expanding/nitinol	Pericardial tissue	NA	TF

PD: pericardium; NA: not applicable; TF: transfemoral; TA: transapical; TJ: transjugular.

## Data Availability

Not applicable.
